# A Facile Electrochemical Sensor Labeled by Ferrocenoyl Cysteine Conjugate for the Detection of Nitrite in Pickle Juice

**DOI:** 10.3390/s19020268

**Published:** 2019-01-11

**Authors:** Xiao-Zhen Feng, Annaleizle Ferranco, Xiaorui Su, Zhencheng Chen, Zhiliang Jiang, Guo-Cheng Han

**Affiliations:** 1 Key Laboratory of Ecology of Rare and Endangered Species and Environmental Protection (Guangxi Normal University), Ministry Education, Guangxi Key Laboratory of Environmental Pollution Control Theory and Technology, Guilin 541004, China; fxz97118@guet.edu.cn; 2 School of Life and Environmental Sciences, Guilin University of Electronic Technology, Guilin 541004, China; 18137725539@163.com (X.S.); chenzhcheng@163.com (Z.C.); 3 Department of Physical and Environmental Sciences, University of Toronto Scarborough, Toronto, ON M1C 1A4, Canada; annaleizle.ferranco@gmail.com

**Keywords:** electrochemical sensor, nitrite, ferrocenoyl cysteine, screen-printed electrode, disposable

## Abstract

Simple and facile electrochemical sensors for nitrite detection were fabricated by directly depositing ferrocenoyl cysteine conjugates Fc[CO-Cys(Trt)-OMe]_2_ [Fc(Cys)_2_] or Fc[CO-Glu-Cys-Gly-OH] [Fc-ECG] on screen-printed electrodes (SPEs). The modified carbon electrodes were characterized by scanning electron microscopy (SEM), cyclic voltammetry (CV) and differential pulse voltammetry (DPV). Results indicated that Fc-ECG/SPE sensor showed enhanced current response and a lower overpotential than Fc(Cys)_2_/SPE sensor for nitrite detection. Optimal operating conditions were estimated for nitrite detection by DPV. The concentration of nitrite showed a good linear relationship with the current response in the range of 1.0–50 μmol·L^−1^ and with 0.3 μmol·L^−1^ as the concentration for limit of detection. There were no interferences from most common ions. The development of this electrochemical sensor was used for nitrite detection in pickled juice with a R.S.D. lower than 2.1% and average recovery lower than 101.5%, which indicated that disposable electrochemical sensor system can be applied for rapid and precise nitrite detection in foods.

## 1. Introduction

Nitrite is a good antimicrobial agent for the prevention of fresh meat and fish products from natural degradation in food preservation [1]. However, nitrite can interact with amines or the amino acids of proteins, especially in cured meat, to form toxic and carcinogenic nitrosamines that may lead to gastrointestinal tumors and stomach cancer [2]. Moreover, high concentrations of nitrite in the human body increases the irreversible oxidation of hemoglobin to methemoglobin, which limits the oxygen loading ability of the blood and is hazardous to human health [3]. It is also a big world problem that nitrite contamination of drinking water in reservoirs and aquifers directly influence human health, which are contributed by agricultural fertilizers and manure. Consequently, accurate quantitative analysis and effective removal of nitrite is of great importance for human health and environmental protection [4,5]. 

Several methods have been employed for nitrite detection, such as chromatography [6], spectrophotometry [7], capillary electrophoresis [8], chemiluminescence [9], colorimetry [10], fluorescence spectroscopy [11], high-performance liquid chromatography [12], and electrochemical methods [13,14]. Electrochemical methods are very popular because of their simple operation, sensitive response and fast detection [15]. The main mechanism for nitrite electrochemical detection is based on reduction or oxidation of nitrite on the electrodes [16]. The oxidation of nitrite on bare electrodes often occurs at high potentials and the electrodes can be poisoned by the species produced in the electrochemical reaction [17]. An appropriate catalyst-modified electrode provides an effective method to lower nitrite potentials [18,19]. Moreover, an optimal mediator may improve the sensitivity and selectivity of modified electrodes for nitrite detection. 

It should be mentioned that the rapid growth of organometallic chemistry has benefitted from the discovery of ferrocene and its derivatives [20]. The most important application of ferrocene and its derivatives is that they can be used as electrochemical probes for detection of a wide range of biomolecules with great stability in aqueous media. The well-characterized one-electron reversible oxidation wave of ferrocene contributes to the development of electrochemical sensors [21,22,23]. Moreover, ferrocene derivatives are suitable recognition receptors, and interact with various enzymes, DNA, RNA and other macromolecular substances in cells, which can be monitored by their good redox properties [24,25,26]. 

In this study, simple and facile electrochemical sensors were developed by directly depositing ferrocene derivatives Fc[CO-Cys(Trt)-OMe]_2_ [Fc(Cys)_2_] or Fc[CO-Glu-Cys-Gly-OH] [Fc-ECG] on screen-printed electrodes (SPEs). Two ferrocenoyl cysteine conjugate sensors, Fc(Cys)_2_/SPE and Fc-ECG/SPE, were successfully prepared and characterized. However, Fc-ECG/SPE sensor that was used for nitrite detection showed an enhanced current response and a lower overpotential than Fc(Cys)_2_/SPE sensor, which could be used as an effective sensor for the assessment of nitrite concentration. Moreover, the proposed method was also used to determine the presence of nitrite in pickled juice with satisfactory results. 

## 2. Materials and Methods

### 2.1. Chemicals and Reagents

Sodium nitrite was purchased from Aladdin Reagent Co. Ltd. (Shanghai, China). Fc[CO-Cys(Trt)-OMe]_2_ (abbreviated as Fc(Cys)_2_) was synthesized according to [27]. Fc[CO-Glu-Cys-Gly-OH] (Fc-ECG) was purchased from Shanghai Chang Xi Biotechnology Co. Ltd. (Shanghai, China). Disodium hydrogen phosphate (Na_2_HPO_4_), sodium dihydrogen phosphate dehydrate (NaH_2_PO_4_), H_3_PO_4_ and NaOH were purchased from Guilin Xinpeng Chemical Reagent Sales Department (Guilin, China). Pickled mustard was purchased from local supermarket, the pickle juice was diluted directly by PBS for test after centrifugal separation from pickled mustard. All other reagents were of analytical grade and used without further purification. All aqueous solutions were prepared with Milli-Q water (Millipore, Burlington, MA, USA).

### 2.2. Preparation of Fc(Cys)_2_/SPE and Fc-ECG/SPE Electrochemical Sensors

**Figure 1:**
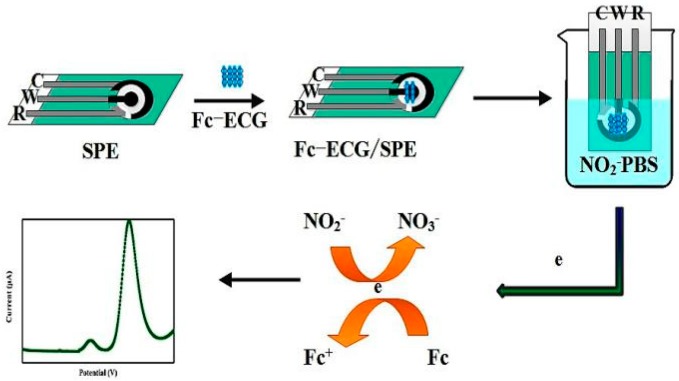
Fabrication of Fc-ECG/SPE electrochemical sensor for nitrite detection by DPV.

At first, 0.5 mmol·L^−1^ of Fc(Cys)_2_ stock solution was prepared by dissolving Fc(Cys)_2_ in acetonitrile solvent. 0.5 mmol·L^−1^ Fc-ECG stock solution was prepared by dissolving Fc-ECG in Milli-Q water. 10.0 mmol·L^−1^ nitrite stock solution was prepared by dissolving sodium nitrite in Milli-Q water. 0.1 mol·L^−1^ phosphate buffer solutions (PBS) with various pH values were used as the supporting electrolyte, and prepared by mixing 0.1 mol·L^−1^ NaH_2_PO_4_ and 0.1 mol·L^−1^ Na_2_HPO_4_. The pH value of PBS was adjusted by 0.1 mol·L^−1^ of H_3_PO_4_ and 0.1 mol·L^−1^ of NaOH solution. The prepared solutions were stored in a refrigerator at 4 °C. All stock solutions were diluted to the appropriate concentration before experiment, including separated pickle juice directly diluted by PBS. Then, the fabrication of the sensors were carried out by depositing either electroactive material Fc(Cys)_2_ or Fc-ECG onto the surface of the SPE for 30 min until dry, and electrochemical sensors of Fc(Cys)_2_/SPE and Fc-ECG/SPE for nitrite detection by DPV were prepared. The preparation of electrochemical sensor of Fc-ECG/SPE for nitrite detection by DPV is simple and described in Figure 1.

### 2.3. Apparatus and Electrochemical Performance

Morphologies of the electrochemical sensors were analyzed by field emission scanning electron microscope produced from Hitachi High-Technologies Corporation (Tokyo, Japan) at 3 kV and 10,100 nA with the working distance of 6000 μm.

The electrochemical analyses were carried out using screen-printed electrodes that were provided by Nanjing Yunyou Biotechnology Co. Ltd. (Nanjing, China). One modified carbon paste electrode was used as the working electrode (φ 3 mm), another carbon paste electrode as the auxiliary electrode and Ag/AgCl electrode as the reference electrode in an enclosed faraday cage, which were connected to a CHI660E electrochemical workstation (Shanghai Chenhua Instrument Co., Ltd., Shanghai, China). For the cyclic voltammetry (CV) experiments, all measurements were carried out with a scan rate of 100 mV·s^−1^ in range of −400–+1200 mV in 0.1 mol·L^−1^ phosphate buffer (PBS) as the supporting electrolyte. DPVs were performed in 0.1 mol·L^−1^ PBS with a pulse amplitude of 50 mV in range of −400–+1200 mV.

## 3. Results and Discussion

### 3.1. Characterization and Electrochemical Properties of Modified Electrodes

**Figure 2:**
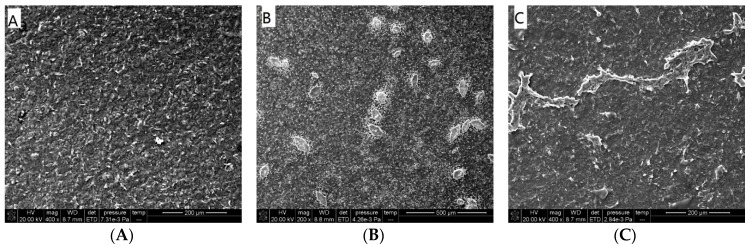
SEM images of different materials modified SPEs ((**A**): naked SPE, (**B**): Fc(Cys)_2_/SPE; (**C**): Fc-ECG/SPE).

After directly dropping electroactive material ferrocenoyl cysteine conjugates of Fc(Cys)_2_ or Fc-ECG SEM was employed to characterize the electrode surface in order to verify the successful electrode modification. Figure 2 shows the SEM images of different materials modified SPEs.

Figure 2A presents a number of carbon particles on the surface of naked SPE with rough amorphous carbon morphology. After depositing eitherelectroactive material Fc(Cys)_2_ or Fc-ECG, the surface of SPE became smooth (Figure 2B,C), leaf spot and spray zones were observed on the surface of electrode, respectively. These figures confirmed that electroactive materials were coated onto the SPE surface successfully. 

**Figure 3:**
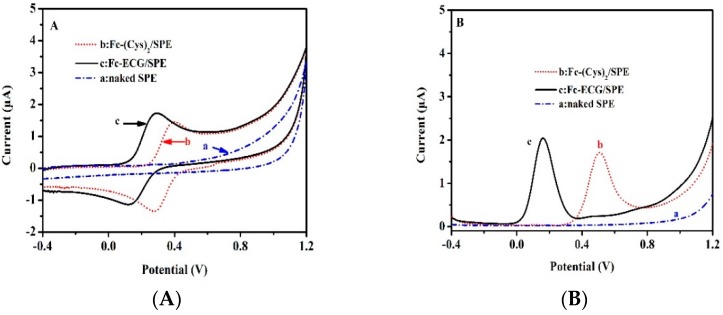
CV (**A**) and DPV (**B**) curves for different materials modified SPEs in 0.1 mol·L^−1^ PBS (pH 7.0) (a: naked SPE, b: Fc(Cys)_2_/SPE; c: Fc-ECG/SPE).

The electro-active response property is important, and should be investigated after morphology characterization. Figure 3 shows CV (A) and DPV (B) curves for different material modified SPEs in PBS (pH 7.0), respectively.

As can be seen in Figure 3A, curve a was a straight line and there was no electrocatalytic response from the naked SPE in PBS. After depositing Fc(Cys)_2_ or Fc-ECG onto the surface of SPE, electro-catalytic response of two modified SPEs were observed in curves b and c, respectively. Fc(Cys)_2_/SPE showed redox peaks at 0.40 and 0.28 V with 1.45 μA and -1.34 μA peak current response, respectively. The Fc-ECG/SPE showed redox peaks at 0.29 and 0.13 V with 1.71 and −1.12 μA peak current response, correspondingly. The electrochemical process indicated a quasi-reversible reaction, for both Fc(Cys)_2_/SPE and Fc-ECG/SPE. Similar results were observed from DPV signals at 0.5 and 0.16 V with peak current response of 1.73 and 2.06 μA, as shown in Figure 3B. All the results illustrated the successful modification of the electrodes by different active electrochemical materials, which was consistent with the SEM results.

### 3.2. Electrochemical Behavior of Nitrite on the Modified Electrode

**Figure 4:**
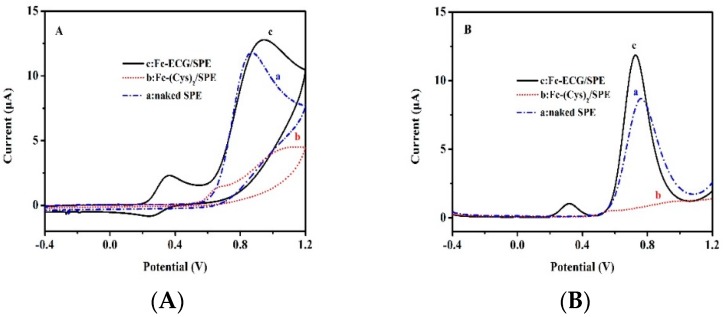
CV (**A**) and DPV (**B**) curves of different materials modified SPEs for detection of 100 μmol·L^−1^ nitrite in 0.1 mol·L^−1^ PBS (pH 7.0) (a: naked SPE, b: Fc(Cys)_2_/SPE; c: Fc-ECG/SPE).

After successful characterization of electrochemical sensors, the actual application was investigated. Figure 4 shows CV (A) and DPV (B) curves of 100 μmol·L^−1^ of nitrite on different modified electrodes in PBS (pH 7.0).

As can be seen in Figure 4A, the oxidation potential for nitrite on the naked SPE, Fc(Cys)_2_/SPE, and Fc-ECG/SPE was 0.86, 1.06, and 0.95 V with peak current response of 11.78, 4.40, and 12.79 μA, respectively. Fc-ECG/SPE can enlarge the current response compared to the naked SPE and Fc(Cys)_2_/SPE for nitrite oxidation. Only one unobvious oxidation peak at 0.66 V was observed in the internal signal of Fc(Cys)_2_. Fc-ECG showed its identified internal redox peaks at 0.36 and 0.26 V with a weak peak current response. We can observe the similar results from the DPV experiments as shown in Figure 4B. The oxidation potential for nitrite on the naked SPE and Fc-ECG/SPE were 0.76 and 0.73 V with peak current of 8.64 and 11.88 μA in PBS (pH 7.0), respectively. Fc-ECG/SPE sensor not only can enlarge the current response by 37.5%, but can also lower the oxidation overpotential from 0.76 to 0.73 V. Moreover, it shows the identified internal oxidation peak at 0.32 V with a weak oxidation peak current response. However, no potential and current response were observed in Fc(Cys)_2_ and nitrite oxidation on the SPE surface. All the results showed that nitrite oxidation possessed a lower overpotential on Fc-ECG/SPE sensor than other modified electrodes, which was also demonstrated by our previous work of hemoglobin catalysis [28]. It presents excellent electrochemical catalytic ability for further study, which can potentially be used for nitrite detection. 

### 3.3. Optimization of Experimental Parameters

**Figure 5:**
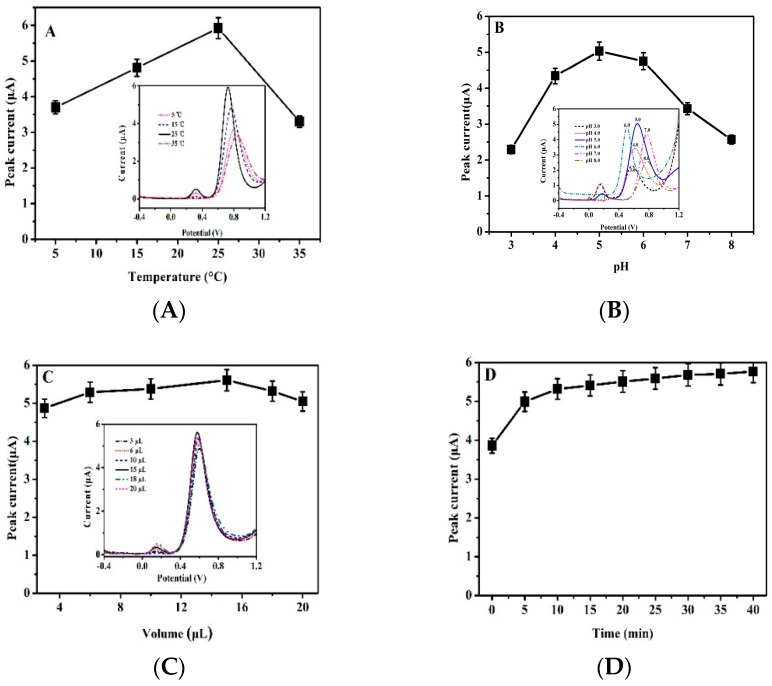
Current responses (The inserts are DPV curves) for 50 μmol·L^−1^ nitrite oxidation on Fc-ECG/SPE sensor in 0.1 mol·L^−1^ PBS with a pulse amplitude of 50 mV. (**A**): pH 7.0, at 5, 15, 25 and 35 °C; (**B**): pH 3.0, 4.0, 5.0, 6.0, 7.0, 8.0, at 25 °C; (**C**): pH 5.0, with 3, 6, 10, 15, 18, 20 μL of Fc-ECG on electrode at 25 °C; (**D**): pH 5.0, with 0-40 min reaction time at 25 °C.

The optimization of experimental parameters, including detection temperature, pH of phosphate buffer solution, drop volume of electroactive material and deposition time, wereinvestigated. Figure 5A shows the DPV curves for nitrite oxidation on Fc-ECG/SPE sensor at different temperatures. Figure 5B,C show the current response of different pH values and different drop volume of Fc-ECG electroactive material. Figure 4D expresses the current response of reaction time on the electrode surface for nitrite oxidation.

As can be seen in Figure 5A, the electrochemical sensor of Fc-ECG/SPE displayed good electro-catalytic activities for nitrite at 25 °C, and 25 °C (room temperature) was chosen for the following experiments. The pH value is also an important factor for nitrite detection. The Fc-ECG/SPE showed the highest current response of oxidation peaks, and the redox peak shift to a lower potential at pH = 5.0, as shown in Figure 5B. The decrease in oxidation peak currents at higher pH (>5) is due to the shortage of protons, which affects the electrocatalytic oxidation of nitrite. There was also a decrease observed when the pH of the solution was below pH 5, indicating that the nitrite ions are not stable in highly acidic environment, the active NO_2_^−^ species would be converted to NO and NO_3_^−^ and form HNO_3_ [29], which can also affect the catalytic reaction process of nitrite oxidation. It may react as following at a lower pH:

2H^+^ + 3NO_2_^−^ → 2NO + NO_3_^−^ + H_2_O (1)

Finally, the maximum oxidation peak current was observed at pH 5, which was selected as the optimal pH and used for further electrochemical studies. The effects of drop volume of the Fc-ECG electroactive material for nitrite oxidation are shown in Figure 5C. It can be clearly seen that the drop volume of electroactive material showed no obvious effect, and 15 μL of Fc-ECG resulted in an adequate sensor response; no further adjustments were made and this volume was for further studies as discussed below. The current increased with increased reaction time, and the current became stable after 20 min, as shown in Figure 5D. Therefore, 20 min was therefore chosen for electrode reaction time. 

### 3.4. Nitrite Detection by Electrochemical Sensors and the Sensitivity, Selectivity, Repeatability and Stability Study

**Figure 6:**
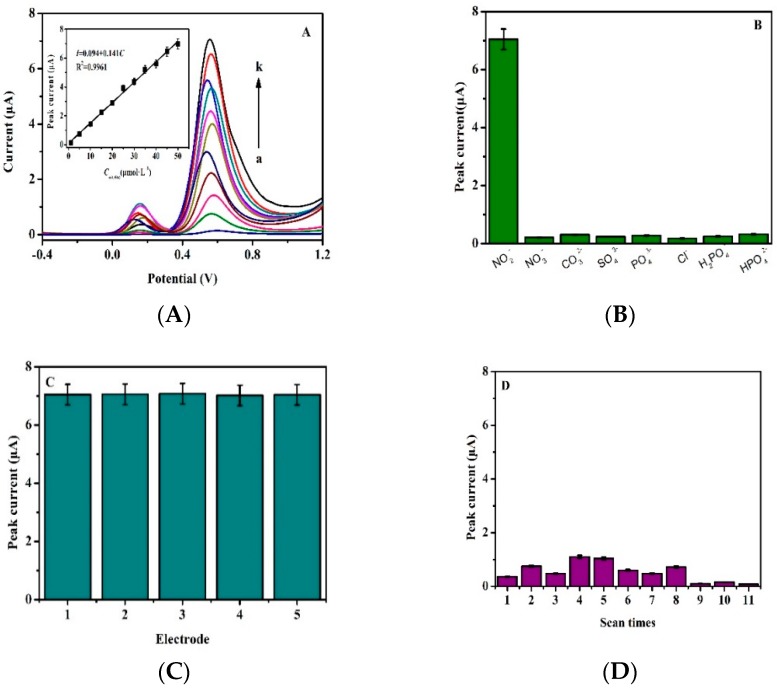
(**A**): DPV for different concentrations of nitrite on Fc-ECG/SPE (a-k: 1.0, 5.0, 10, 15, 20, 25, 30, 35, 40, 45, 50 μmol·L^−1^), insert: calibration plot for various concentrations of nitrite; (**B**): The selectivity of Fc-ECG/SPE sensor for nitrite, NO_3_^−^, CO_3_^2−^, SO_4_^2−^, PO_4_^3−^, Cl^−^, H_2_PO_4_^−^, and HPO_4_^2−^, the concentration of each compound is 100 μmol·L^−1^; (**C**): The repeatability of Fc-ECG/SPE sensor; (**D**): The stability of Fc-ECG/SPE sensor.

As mentioned above, the primary goal of this work was to prepare electrochemical sensors for detection of nitrite by DPV. Figure 6A shows the DPV curves for different concentrations of nitrite on Fc-ECG/SPE sensor in PBS, with a pulse amplitude of 50 mV. Figure 6B–D show the selectivity, repeatability and stability of Fc-ECG/SPE sensor for nitrite oxidation, respectively.

Under optimal conditions, it was observed that the Fc-ECG/SPE sensor not only showed a characteristic peak at 0.16 V without a strong peak current response, but also showed the characteristic peak at 0.55 V versus Ag/AgCl electrode for nitrite oxidation (shown in Figure 6A). However, the oxidation peak of nitrite was up at 0.75–0.85 V on most modified electrodes, as shown in Table 1. Therefore, its oxidation overpotential was reduced deeply with different potential of 210 mV from 0.76 to 0.55 V, because Fc-ECG can act as electron transfer medium and promote charge transfer [28]. These results fully confirmed that Fc-ECG/SPE can be used for nitrite detection based on its excellent electrochemical catalytic ability. The electrochemical oxidation peak currents of nitrite increased with increased concentrations and all followed a linear relationship, as shown in the Figure 6A insert. The peak potentials were kept steady and their calibration curves were linear at concentration ranges of 1.0–50 μmol·L^−1^ for nitrite with detection limits of 0.3 μmol·L^−1^. The linear regression equation for nitrite was expressed as follows: *I* = 0.094 + 0.141 *C* (R^2^ = 0.9961) (*I*: μA; *C*: μmol·L^−1^). 

As an electrochemical sensor, it is important to evaluate its selectivity and stability for actual application [30,31]. To explore the selectivity of the Fc-ECG/SPE, the possible interference factors of nitrite detection were investigated. 100 μmol·L^−1^ of NO_3_^−^, CO_3_^2−^, SO_4_^2−^, PO_4_^3−^, Cl^−^, H_2_PO^4−^, and HPO_4_^2−^ were used to study the selectivity of the sensor. The different types of interfering agents did not show strong selectivity as shown in Figure 6B, indicating that the sensor possessed good selectivity for the detection of nitrite. A comparison of previously reported electrochemical sensors for nitrite detection with this work is shown in Table 1. Compared with other modified electrodes, although the Fc-ECG/SPE sensor did not show a very wide concentration range and low detection limit, it exhibited low overpotential for nitrite oxidation. 

The repeatability was studied by five modified electrodes which were prepared independently. The relative standard deviation (R.S.D) was 0.32% for 50 μmol·L^−1^ nitrite oxidation by comparing the peak currents in 0.1 μmol·L^−1^ PBS (pH 5.0) as shown in Figure 6C, indicating its excellent repeatability. 

On the other hand, the stability of the Fc-ECG/SPE was also investigated. After modifying the electrode, the Fc-ECG/SPE sensor was used for different concentration nitrite detection. As shown in Figure 6D, there was no obvious change in the peak current intensity even after 11 times scans which were obtained from Figure 6A at 0.16 V. Therefore, the proposed electrochemical sensor displayed high selectivity and good stability, and was suitable for nitrite detection.

### 3.5. Real Sample Analysis

To verify the feasibility of Fc-ECG/SPE sensor in practical application, it was applied to detect nitrite concentrations in pickled juice. All the samples were filtrated and diluted with PBS (pH 5.0) to obtain linear concentration range. The analysis data are summarized in Table 2, in which the average recovery of spiked samples was less than 101.5%, and the relative standard deviations (R.S.D.) for the DPV response currents were less than 2.1%, showing the potential practical application of as-prepared sensor in real samples.

## 4. Conclusions

In summary, we have demonstrated a simple strategy for preparing Fc-ECG/SPE sensors for nitrite detection in food samples which were developed by directly depositing the electroactive material Fc-ECG on SPEs by DPV. Electrochemical measurements demonstrated that the Fc-ECG/SPE sensor with its characteristic signal showed improved electrocatalytic performance towards oxidation of nitrite by lowing the overpotential and increasing the peak current, due to the fact that ferrocenoyl cysteine conjugate can act as an electron transfer medium and promote charge transfer. These results demonstrated that Fc-ECG/SPE sensor can be a promising electrochemical sensor for electrocatalytic applications towards nitrite oxidation in the range of 1.0–50 μmol·L^−1^ with 0.3 μmol·L^−1^ limit of detection in pickle juice with a R.S.D. lower than 2.1% and average recovery lower than 101.5%, which was proved to be applicable for nitrite detection in pickle juice with good selectivity. 

## Figures and Tables

**Table 1:** Comparison of nitrite detection by different materials modified electrodes with direct electrochemistry.

Modified Electrode	Peak Potential (V)	pH	Detection Limit (μmol·L^−1^)	Linear Range (μmol·L^−1^)	Reference
CR-GO/GCE	0.8 ^a^	5.0	1.0	8.9–167	[32]
Pd/SWCNT	0.75 ^a^	4.0	0.25	2–238	[33]
CFO/SPCE	0.8 ^a^	5.0	0.007	0.016–1921	[29]
Ag/Cu/MWNT/GCE	0.85 ^a^	7.0	0.2	1.0–1000	[34]
Dendrimer/AuNPs/GC	0.85 ^b^	5.0	0.2	10–5000	[35]
Ag-PAMAM/GCE	0.8 ^b^	6.0	0.4	4.0–1440	[36]
Hb/Au/GACS/GCE	0.85 ^b^	7.0	0.01	0.05–1000	[37]
Fc-ECG/SPE	0.55 ^a^	5.0	0.3	1.0–50	This work

^a^ Ag/AgCl electrode; ^b^ Saturated calomel electrode.

**Table 2:** Detection of nitrite in pickled juice samples (n = 5).

Analyte	Detected (μmol·L^−1^)	Added (μmol·L^−1^)	Found (μmol·L^−1^)	Average Recovery (%)	RSD (%)
Pickle juice	20.0 ± 0.4	10.0	30.4 ± 0.8	101.4	2.1
		20.0	40.3 ± 0.9	100.9	1.8
